# Pandemics and the precautionary principle: an analysis taking the Swedish Corona Commission’s report as a point of departure

**DOI:** 10.1007/s11019-023-10139-x

**Published:** 2023-02-13

**Authors:** Anders Nordgren

**Affiliations:** Centre for Applied Ethics, Linköping University, 581 83 Linköping, Sweden

**Keywords:** COVID-19, Pandemics, Precautionary principle, Specification, Sweden

## Abstract

In the initial phase of the COVID-19 pandemic, Sweden’s response stood out as an exception. For example, Sweden did not introduce any lockdowns, while many other countries did. In this paper I take the Swedish Corona Commission’s critique of the initial Swedish response as a point of departure for a general analysis of precaution in relation to pandemics. The Commission points out that in contrast to many other countries Sweden did not follow ‘the precautionary principle’. Based on this critique, the Commission proposes that the precautionary principle should be included among Sweden’s guiding principles for crisis management. However, as the debate on this principle during the last 30 years indicates, the principle is loaded with problems. I discuss one of these problems, namely its lack of clarity. I argue, however, that this problem is not unsurmountable. A principle is lacking clarity precisely by being a principle and not a rule with a well-defined meaning. As a principle it indicates a direction but does not prescribe a specific action. However, to be action-guiding its content needs to be specified by rational deliberation. With this in mind, I propose a framework for specification of the precautionary principle as applied to pandemics. The framework focuses on the principle’s four key elements: threat, uncertainty, action and responsibility. I also suggest certain general ethical restrictions on specification.

## Introduction

In the beginning of the COVID-19 pandemic, Sweden’s response stood out as an exception compared to many other countries, including its Nordic neighbours. For example, Sweden did not have any lockdowns. Over time Sweden’s policy became stricter but did still not include any lockdowns. Sweden’s exceptionalism received much attention in international media. At present this is no longer the case and up till now Sweden has had a lower excess mortality than most European countries, except, for example, its neighbouring Nordic countries (The Corona Commission 2022, 1, 7 (abbreviated as ‘CC’ in the following); Coronakommissionen 2022, 225). However, the initial Swedish response is still interesting at a more fundamental level. It raises the question of the role of precaution in responding to pandemics like COVID-19. Actually, this is a key issue in the Swedish Corona Commission’s critique of the initial Swedish policy. According to the Corona Commission, Sweden’s initial shortcomings compared to many other countries were partly due to the fact that Sweden in contrast to these other countries did not follow ‘the precautionary principle’. Based on this critique, the Corona Commission proposes that the precautionary principle should be included among Sweden’s principles for crisis management (CC 2022, 3, 7, 20).

This paper takes the Corona Commission’s report–or, more precisely, the Corona Commission’s critique of Sweden’s initial response to COVID-19–as a point of departure for a general analysis of the precautionary principle as applied to pandemics. The paper consists of three parts. The first part analyses the Corona Commission’s critique and its proposal to include the precautionary principle among Sweden’s guiding principles for responding to pandemics and other crises. The second part investigates a key problem linked to the precautionary principle, namely its lack of clarity, and proposes how to resolve this problem by specification of the principle. The final part proposes a framework for specification of the precautionary principle as applied to pandemics as well as certain general ethical restrictions on specification.

### Case: the Corona Commission’s critique of the initial Swedish response to COVID-19

#### The need for a precautionary principle

The Swedish Corona Commission criticizes Sweden’s initial response to COVID-19 in the following way:

The measures taken were too few and should have come sooner. In February/March 2020, Sweden should have opted for more rigorous and intrusive disease prevention and control measures. In the absence of a plan to protect older people and other at-risk groups, earlier and additional steps should have been taken to try to slow community transmission of the virus (CC 2022, 3).

According to the Corona Commission, Sweden did too little, too late. Sweden was lacking in readiness to protect the elderly and other vulnerable groups. Given this lack of readiness, Sweden should have acted more decisively to slow the spreading of the virus in society. Compared to many other countries–including the other Nordic countries–Sweden acted more slowly and introduced less intrusive measures. This is how the Corona Commission describes these differences:

In so far as it is possible to talk about a kind of “ideological” difference between the Nordic countries in terms of the measures chosen, it has more to do with their response to the precautionary principle’s requirement to act despite incomplete information. The approach chosen by Sweden was based on voluntary measures and personal responsibility, rather than more intrusive interventions … [D]isease prevention and control had been marked by a slowness of response. Our Nordic neighbours and many other countries introduced rigorous measures, such as various forms of lockdown and bans on entry, more or less immediately (CC 2022, 7).

Sweden acted slowly, while many other countries acted promptly. Sweden issued recommendations, while many other countries issued legal regulations. It undertook voluntary measures and emphasized personal responsibility, while many other countries introduced lockdowns and bans on entry. The Corona Commission explains in part the slowness by the fact that Sweden did not follow ‘the precautionary principle’. It understands this principle in the following way:

The Commission returns several times to discussions of the ‘precautionary principle’. This can be seen as a basic attitude in responding to a threatening situation when the information available is highly uncertain and incomplete. The principle implies that, in such situations, decision-makers should not passively wait for a better understanding, but actively take steps to counter the threat. It means, in other words, that it is better to act than to wait for better data for decision-making … (CC 2022, 3).

We notice here that the Corona Commission’s definition (for a reconstructed version, see below) includes four elements that explicitly or implicitly are common in various formulations of the principle (cf. Sandin 1999):

- threat: “a threatening situation”,
- uncertainty: “the information available is highly uncertain and incomplete”,
- action: “not passively wait for a better understanding, but actively take steps to counter the threat”, and
- responsibility (Sandin 1999 uses the term “command”): “decision-makers should…”.

Rather than grounding their policy on the precautionary principle, Swedish decision-makers followed the *Communicable Disease Act* which stresses evidence-based decision-making. Decisions should be based on “science and proven experience” (CC 2022, 7, 18). It seems that due to this Act decision-makers tended to “passively wait for a better understanding” rather than “actively take steps to counter the threat”.

Another contributing factor behind Sweden’s slow response was the decentralized responsibility in the Swedish governmental system (CC 2022, 12). Central government (the Government, ministries, agencies) and local government (local authorities, regions) have different responsibilities. Moreover, ministries and agencies are independent of each other and have different responsibilities (CC 2022, 14). Furthermore, among its principles for crisis management Sweden has included ‘the responsibility principle’. This principle states that those normally responsible for a societal activity should continue to be so also in times of crises (CC 2022, 14; Coronakommissionen 2022, 291–292). This decentralized responsibility led to less effective overall leadership and a lack of over-arching responsibility for responding promptly to COVID-19.

Based on this critique, the Corona Commission proposes that Sweden should include a ‘precautionary principle’ among its principles for crisis management to be applied in future pandemics. It also proposes a more centralized governmental system with “a body providing clear national crisis leadership” reporting directly to the Government (CC 2022, 20–21).

Focusing on the precautionary principle, what would this principle have implied for the management of the pandemic in its initial stage? Here are some examples. As I interpret the Commission, Sweden should have immediately stopped import of the virus by testing and contact-tracing of travellers returning from winter sports breaks in Northern Italy and limited spreading in society by temporary closures of indoor settings like restaurants, shopping centres and sports events as well as by a temporary ban on entry to the country. Moreover, The Public Health Agency should have promoted the use of protective masks rather than dismissed it (CC 2022, 8–10). However, the Commission does not propose that Sweden should have introduced lockdowns as many other countries did:

In light of current knowledge, however, the Commission is not convinced that extended or recurring lockdowns, as introduced in many other countries, are a necessary element in the response to a new, serious pandemic outbreak (CC 2022, 9).

The Commission presents three reasons. First, many countries introducing lockdowns have experienced much worse outcomes than Sweden. Second, lockdowns restrict citizens’ freedom in an unjustifiable way. Third, lockdown policies do not seem sustainable in the long term and may lead to protests–sometimes violent–which has been the case in many countries (CC 2022, 9).

#### Comments on the Corona Commission’s proposal

Two remarks on the Corona Commission’s proposal to include a ‘precautionary principle’ among its principles for crisis management seem warranted. First, the Corona Commission shows no awareness of the multitude of versions of the precautionary principle proposed in a variety of areas such as environmental protection, sustainable development, human health, food safety and trade (for example, United Nations 1992; The Wingspread Statement 1998; Commission of the European Communities 2000; Secretariat of the Convention on Biological Diversity, 2000). This is serious, since the formulation of the principle proposed by the Corona Commission is not self-evident. Other versions of the principle might have informed the proposal in a constructive way. In particular, it is serious that the Corona Commission neglects to explore the interpretation and legal status of the precautionary principle in countries that responded to the COVID-19 pandemics on the basis of this principle. Most importantly, the Corona Commission neglects to mention the significance and central place of the precautionary principle in the EU legislation (Commission of the European Communities, 2000; Fisher 2002; Meßerschmidt 2020; Goldner Lang 2021).

Second, the Corona Commission shows no awareness of the multidisciplinary debate on the precautionary principle during the last 30 years and the problems linked to the principle pointed out in this debate. The principle is not as simple and obvious as the Corona Commission seems to assume. It has been argued, for example, that the principle is incoherent, lacking clarity, redundant, implying a risk for unfounded panic and impeding scientific and technological development (for the debate on these issues, see for example, Fisher 2002; Sandin 2004; Turner and Hartzell 2004; Resnik 2004; Sunstein 2005; Peterson 2006; Arcuri 2007; Randall 2011; Munthe 2011; Hansson 2013; Steel 2014; Meßerschmidt 2020; Birch 2021).

The plurality of versions of the precautionary principle together with the problems pointed out by critics indicate that the Corona Commission’s proposal is theoretically unsatisfying. A more thorough analysis of the problems of the principle as applied to pandemics is warranted. However, this paper is not the place for such an analysis. Below I will rather focus on one key problem among those mentioned, namely its lack of clarity. This problem is illustrated in an excellent way by the difference between the Corona Commission’s proposed application of the principle and the actual application of it by many countries other than Sweden during the COVID-19 pandemic. Based on the principle the Corona Commission states that Sweden should have promptly undertaken more rigorous measures than those initially undertaken, for example, temporary closures of indoor settings and a temporary ban on entry to the country. However, it does not propose any lockdowns. Many other countries, on the other hand, did initiate lockdowns for precautionary reasons. So, the precautionary principle can be understood and applied in different and even conflicting ways. How should we understand this lack of clarity and how should this lack of clarity be resolved?

I will argue that the lack of clarity of the precautionary principle is not an unsurmountable problem. I proceed in three steps. In the first step I suggest that the precautionary principle as a principle may have a value despite its lack of clarity. In the second step I argue that to be action-guiding it needs to be specified and that specifying is a matter of rational deliberation. In the third step I present a framework for specification of the precautionary principle as applied to pandemics. This framework can be seen as a tool for improving the application of the precautionary principle to future pandemics. I also present certain general ethical restrictions on specification.

### Lack of clarity and the need for specification

#### A principle is not a well-defined rule

It is vital to understand the true nature of principles like the precautionary principle. Often criticism of the precautionary principle seems to be based on a misunderstanding of what it means for a principle to be a principle. As Fisher argues:

Much of the discussion concerning the precautionary principle has proceeded on the assumption that the principle is a ‘bright line’ autonomous rule that dictates a particular outcome in a certain set of circumstances. As such, the principle is understood as analogous to rules such as those that state that ‘if you drive over 60 kmph you have broken the law and will be fined 100 euros’. … Yet, these critiques, by assuming the principle is a rule, proceed on the wrong basis and ignore a basic feature of the principle–that it is, in legal terms, a principle rather than a rule. While the concept of ‘principle’ will vary depending on its jurisprudential and jurisdictional context, in all cases a principle is not an ‘explicitly formulated’ rule that is unchanging in its application. Rather its application is flexible and will depend on specific circumstances. Moreover, just as other principles do, it ‘states a reason that argues in one direction, but does not necessitate a particular decision’ (Fisher 2002; footnote numbers excluded).

So, a principle is lacking clarity precisely by being a principle and not a rule. It is not a rule with a well-defined meaning. It indicates a direction but does not prescribe a specific action. To be action-guiding the principle needs to be specified and thus transformed into a rule that prescribes a specific action in a specific context and in a specific set of circumstances. It should be noted that Fisher focuses on the precautionary principle as a legal principle. However, the same need for specification is obvious if it is interpreted as a moral principle or if it refers to a moral dimension of a legal principle.

#### Moral imagination, rational deliberation and constructive dialogue

Lacking clarity is not necessarily a weakness of the precautionary principle. According to Turner and Hartzell, this unclarity gives it a rhetorical force (2004). However, I would rather emphasize that, although unclear, the principle may be a useful starting-point for decision-makers in a non-ideal situation characterized by serious threat and uncertainty. By indicating a direction the principle may in a constructive way be subject to moral imagination. Moral imagination may suggest new possibilities in a new and threatening situation. It implies envisioning a great variety of options for framing the situation and a great variety of options for action within this situation (Nordgren 1998). In the next step this may lead to rational deliberation and constructive dialogue on how to specify the principle and thereby provide action-guiding content, including support for political decisions and legal regulations.

#### Providing action-guiding content by specification

The precautionary principle needs to be specified, that is, implemented and operationalized in moral rules, legal regulations and policies. How should this specification be carried out? A promising option is the ‘model of specification’ suggested by Richardson (1990). This model is not without problems (Rauprich 2011), however these problems will not bother us here. This is how Richardson presents this model:

The central assertion of the model of specification is that specifying our norms is the most important aspect of resolving concrete ethical problems, so that once our norms are adequately specified for a given context, it will be sufficiently obvious what ought to be done (Richardson 1990).

Here are some of the key features of this model of specification.

- The norms that can be specified are only those that are not formally absolute (“for all actions x”) but hold merely generally (“for most actions x”).
- The norm to be specified and the specification of it must be of the same normative type, for example, a permission or a requirement.
- The specification qualifies the original norm by adding relevant statements about, for example, what, when, by what means and by whom.
- The specification is a result of rational deliberation.
- The specification is open for revision by further rational deliberation.
- The specification is not to be considered a new norm but precisely a specification of the original norm since the absolute counterpart of the specification (“for all actions x) would be an instance of the absolute counterpart of the original norm (“for all actions x”) (Richardson 1990).

Richardson’s model is designed for specification of moral norms. I suggest, however, that it is applicable also to the moral dimension of legal norms and to legal norms as such.

As indicated, it is important to distinguish a specification of a principle from an improved formulation of a principle. A specification of a principle is a norm that gives the principle more action-guiding content. In line with the third point above, a specification qualifies the principle by adding statements about what, when, by what means and by whom. Increased precision is achieved by eliminating or at least reducing ambiguity and vagueness. The specification has a special logical relation to the principle: the absolute counterpart of the specification would be an instance of the absolute counterpart of the original norm, in this case the principle. In contrast, an improved formulation of a principle is merely a new formulation of the principle, even if it in some respects is considered to have somewhat better precision than the original principle, for example by being explicitly restricted to pandemics rather than being more general and including also the development and use of new technology. For this reason it might be better to talk about several different precautionary principles rather than a single one (Hartzell-Nichols 2013). However, as is commonly the case in the debate I will still below talk about different formulations of ‘the’ precautionary principle rather than different precautionary principles. The focus is on the precautionary principle as applied to pandemics, if not stated otherwise.

Is it possible to interpret the Corona Commission’s concrete proposals concerning precautionary measures–including the rejection of lockdowns–as parts of a specification of its version of the precautionary principle? Yes, it can probably be reconstructed in this way, but it is not presented as such in the Corona Commission’s report. To be a specification the concrete proposals must be presented systematically and explicitly as parts of a specification of the principle.

### A framework for specification of the precautionary principle as applied to pandemics

Specification of the precautionary principle as applied to pandemics requires dealing with the lack of clarity by eliminating or at least reducing ambiguity and vagueness. Eliminating or reducing ambiguity is a matter of clarifying which type of x is of concern among many possible types of x, for example one type of threat or action among various types of threats or actions. Eliminating or reducing vagueness is a matter of clarifying what degree of x is sufficient for taking action or what degree of x is normatively acceptable. An example is clarifying that a certain degree of seriousness of a threat is sufficient for taking action while a lower degree of seriousness is not. Another example is clarifying what is a normatively acceptable degree of intrusiveness of action in contrast to an unacceptable degree.

Below I propose a framework for dealing with problems of specification. It is intended to be a tool for moral imagination, that is, for investigating various options for specification. By using the framework, the application of the precautionary principle to future pandemics might be improved (see Table 1).

Let us begin with a brief look at two versions of the precautionary principle.

#### Two versions of the precautionary principle

The definition of the precautionary principle proposed by the Corona Commission can be reconstructed as follows (CC 2022, 3):

In responding to a threatening situation when the information available is highly uncertain and incomplete, decision-makers should not passively wait for a better understanding but actively take steps to counter the threat.

This definition can be compared to the often cited definition proposed by *The Wingspread Statement on the Precautionary Principle* from 1998:

Where an activity raises threats of harm to the environment or human health, precautionary measures should be taken even if some cause and effect relationships are not fully established scientifically (The Wingspread Statement 1998).

Also in this definition we find the four key elements pointed out above concerning the proposal by the Corona Commission:

- threat: “where an activity raises threats of harm”,
- uncertainty: “even if some cause and effect relationships are not fully established scientifically”,
- action: “precautionary measures”, and
- responsibility: “should be taken…”.

*The Wingspread Statement* focuses mainly on threats to the environment and human health such as “the release and use of toxic substances, resource exploitation, and physical alterations of the environment.” However, it also broadens the scope and stresses that a precautionary approach must be adopted to “all human endeavours” (The Wingspread Statement 1998). This opens for an application also to pandemics, since the spreading of a virus may depend on human action.

The versions of the precautionary principle proposed by the Corona Commission and *The Wingspread Statement* are only examples. As pointed out, many other versions exist. Below I do not presume any particular definition. I only presume that the definition is applicable to pandemics and that it includes these four key elements one way or another.

#### Specifying ‘threat’

The element of threat may lack clarity in several respects. It may be ambiguity concerning type of threat or type of causes of threat. It may be vagueness concerning seriousness or urgency of threat. In specifying the precautionary principle the ambiguity and vagueness need to be eliminated or at least reduced.

##### Type of threat

The formulation by the Swedish Corona Commission mentioned above is completely open and does not clarify what is to count as a threat of harm. However, the context shows that it is intended to apply to societal crises of different kinds, including pandemics (CC 2022, 20). The well-known *Rio Declaration* has a different and more narrow understanding and focuses only on threat of harm to the environment (United Nations 1992). *The Wingspread Statement* broadens the perspective and focuses on threats of harm not only to the environment but also to human health (1998). However, in specifying the type of threat in the case of pandemics it is necessary to talk not just about a threat to human health but to human health on a societal and potentially global scale in contrast to human health on a smaller scale.

##### Type of cause

*The Wingspread Statement* pinpoints human ‘activity’ as the cause of threat (1998). This could, for example, be the development and use of new technology that may pose a risk to the environment or human health. Much discussion of the precautionary principle during the last 30 years has focused on such risky technology, not least biotechnology. The Corona Commission, on the other hand, does not talk about human activity but about a threatening ‘situation’ (CC 2022, 3). This opens for including non-human causes, for example dangerous pathogens (viruses or bacteria). It should be noted, however, that it is possible to describe a pandemic as partly caused by human activity–the focus of *The Wingspread Statement*–since human activity may contribute to the spreading of a pathogen in society. Moreover, a dangerous pathogen may be the result of bioengineering.

##### Threshold for seriousness

Not all threats require precaution in the sense of the precautionary principle. The threat must have a certain degree of seriousness. However, the threshold is unclear and requires specification. In the case of pandemics, the mere designation ‘pandemics’ indicate that the threat is above the threshold, but at the initial stage the seriousness might still be unclear. The contagiousness and the risk for death or serious disease are not yet known. However, the specification needs to clarify a threshold of seriousness at which taking action is mandatory or permitted.

##### Threshold for urgency

The urgency of a threat is another important aspect. Urgency is implicit in all formulations since the point of the principle is to act despite incomplete evidence. In case of a pandemics, the level of urgency depends on the speed of spreading of the pathogen. A threshold for the urgency needs to be specified. At what level of urgency is it mandatory or permitted to take action?

#### Specifying ‘uncertainty’

The element of uncertainty is vague in several respects. Specifying this element is particularly difficult, since it indicates precisely that the decisions are to be made more or less ‘in darkness’, that is, without sufficient evidence.

##### Uncertainty about threat

 For a completely new threat such as a new kind of pandemic, the probability ranges are excessively wide. Available data may not support the assignment of numerical values. The risk will ultimately be determined on the basis of subjective assessments (Birch 2021). The Corona Commission stresses that action should be taken even if the information about the threat is “highly uncertain and incomplete” (CC 2022, 3). *The Wingspread Statement* stresses that action should be taken “even if some cause and effect relationships are not fully established scientifically” (The Wingspread Statement 1998). This indicates that the threshold is set higher by *The Wingspread Statement* than by the Corona Commission. How low should the threshold be for the precautionary principle to be applicable? One way of handling the uncertainty of a threat is to construct a number of scenarios representing various degrees of seriousness and urgency. This was done in many countries during the COVID-19 pandemic. Scenarios may play an important role in framing the situation and be a basis for action. However, at the early stages of a pandemic the probabilities for the various scenarios will be extremely difficult, if not impossible, to estimate. Predictions can hardly be made, although this might change as more knowledge emerges. To handle the uncertainty at the initial stage of a pandemic some might argue that decision-makers due to the precautionary principle should choose the worst-case scenario. Others would, on the other hand, stress that the measures should never be excessive.

##### Uncertainty about effects of action

Uncertainty about short-term and long-term effects may concern not only the threat but also the precautionary measures. In case of a pandemic, the measures might include lockdowns or use of protective masks. If the goal is to respond proportionately to the threat, this uncertainty is a serious problem. When the seriousness of a threat is uncertain, it is difficult to determine a proportionate response. Moreover, the potential effects of action do not only include public health effects but also macroeconomic effects and effects on the public trust in governmental authorities. These uncertainties may be no less than the uncertainty about the pandemic as such (see further below).

#### Specifying ‘action’

Specification of the element of action is of key significance in providing action-guiding content to the precautionary principle. Formulations of the principle often do not clarify what to do other than to undertake ‘precautionary measures’. Specification of ‘action’ means clarifying exactly which precautionary measures decision-makers should undertake or are allowed to undertake.

##### Type of goal for action

In the case of a pandemic, the goals of precaution could be prevention or mitigation of harm.

##### Type of action

To attain the goals of prevention or mitigation many different types of action can be taken, for example, lockdown, ban on entry to the country, use of protective masks or closure of indoor settings such as shopping centres or sports events. The various types of action need to be specified in detail.

##### Normative status of action

A specification of a response to an emerging pandemic needs to be clear on the normative status of various actions. A specification may state that an action is, for example, permitted, recommended, required or prohibited. Moreover, a specification may be considered a legal norm, a moral norm, a moral dimension of a legal norm, a policy statement or a decision rule (in decision theory).

##### Degree of intrusiveness of action

Actions may vary in intrusiveness, that is, violation of privacy or personal freedom. This is obvious in the case of a pandemic. If we illustrate the options by a spectrum, we have intrusive measures such as lockdowns at one extreme and voluntary measures at the other. In the case analysis above, we saw that the precautionary principle has been referred to both in favour of approaches that include lockdowns and in favour of approaches that exclude lockdowns. Another important aspect of intrusiveness is duration. For how long should the measures continue? This is a key issue in the case of pandemics. Rigorous measures that continue to hold for a long time are probably conceived as more intrusive than those that hold only for a short time. The specification of intrusiveness is closely related to the issue of proportionality, which is the next aspect.

##### Degree of proportionality of action

Some versions of the precautionary principle include a requirement of proportionality (Birch 2021), others don’t (The Wingspread Statement 1998). The Corona Commission does not include proportionality in its definition but mentions it by reference to the *Communicable Disease Act* and stresses its relevance for the principle (CC 2022, 7). In the *Communication* on the precautionary principle by the European Commission, proportionality is treated as a requirement to be satisfied by precautionary measures (Commission of the European Communities 2000; Meßerschmidt 2020; Goldner Lang 2021). Birch uses the expression “proportionate precautions” and argues that precautions should be “non-excessive” (Birch 2021). Others may argue that it is better to do too much than too little, that is, ‘better safe than sorry’. In considering proportionality concerning pandemic measures decision-makers in liberal democracies may need to take into account also legal rights and macroeconomic aspects, not merely public health aspects (see further below).

##### Degree of promptness of action

Actions may vary in promptness. Both the proposal by the Corona Commission and *The Wingspread Statement* imply that action should be taken despite uncertainty, but they differ on how promptly. The Corona Commission stresses that decision-makers should “not passively wait” but “actively take steps”. The emphasis is on acting promptly, that is, on acting sooner rather than later (CC 2022, 25). *The Wingspread Statement*, on the other hand, lacks such emphasis. In case of a pandemic, what is considered a reasonable level of promptness depends on the level of urgency of the threat, that is, on the speed of the spreading of the pathogen.

#### Specifying ‘responsibility’

The final element of the precautionary principle concerns the responsibility of decision-makers. This element has five main aspects to be specified: whether decision-makers are under obligation to act under uncertainty or are merely allowed to do so, who the decision-makers more precisely are and how much responsibility they have, the practical procedure for specification, how to resolve conflicts concerning specification, and the obligation or permission to revise decisions and procedures as more evidence emerges.

##### Obligation or permission for decision-makers to act

It is common to distinguish strong and weak versions of the precautionary principle. According to strong versions, decision-makers have an obligation to act despite uncertain and incomplete knowledge. Examples are *The Wingspread Statement* (1998) but also the proposal by the Corona Commission. Weak versions, on the other hand, only state that decision-makers are allowed to act in case of uncertain and incomplete knowledge. An example is the *Rio Declaration* (United Nations 1992). Moreover, it is vital to distinguish the obligation or permission to act despite uncertainty and the normative type of the action to be initiated. According to strong versions, decision-makers may have an obligation to recommend as well as prohibit certain activities. According to weak versions, decision-makers may be allowed to recommend as well as prohibit certain activities. In specifying the response to a pandemic all this must be clarified.

##### Distribution of responsibility

Who are responsible to act? How much responsibility do they have? How centralized or decentralized should responsibility be? This can be clarified in the specification of the principle or in a separate statement. As we saw in the case analysis above, the Corona Commission pointed out that the decentralized responsibility in Sweden’s governmental system was a contributing factor to some of the shortcomings of Sweden in the initial phase of the COVID-19 pandemic. It proposed a more centralized governmental system with a body that should provide clear crisis leadership on a national level and report directly to the Government.

##### Specification through procedure

How reach a decision on which specification of the precautionary principle should be action-guiding? How should the problems of specification be resolved in practice? The answers to these questions are intimately connected to the type of governmental system in which the specification is supposed to be carried out, whether liberal democratic, authoritarian or totalitarian. However, even if we focus on liberal democracies there is a spectrum of governmental systems with varying procedures from highly centralized systems to highly decentralized.

##### Conflicts concerning specification

Within a particular governmental context there may emerge conflicting proposals concerning specification of the precautionary principle as applied to pandemics. How should such conflicts be resolved? In a liberal democracy at least four tools are available. The first three are the same as the tools for specification mentioned above: moral imagination to envision the variety of options (including those proposed by opponents), rational deliberation to assess arguments, and constructive dialogue to reach consensus. If these tools do not lead to a satisfying result, a fourth tool remains, namely voting to reach a majority decision. In the initial phase of a pandemic, the urgency to act makes it extremely important that conflicts concerning specification of the precautionary principle are resolved rapidly.

##### Revision

In the light of emerging evidence, precautionary measures might need to be recalibrated. As the initial phase of a pandemic ends, the precautionary approach may be replaced by a more evidence-based approach. Revision may concern the content of the specification of the precautionary principle as well as the procedure of specification. Flexibility is of key importance under the precautionary principle (Hartzell 2013).

**Table 1:** Framework for specification of the precautionary principle as a applied to pandemics (‘X’ means ‘this is a central aspect of specification’)

Element of the precautionary principle	Aspect of element	Specification: elimination or reduction of ambiguity	Specification: elimination or reduction of vagueness
Threat	Type	X	
	Cause	X	
	Seriousness		X
	Urgency		X
Uncertainty	Threat		X
	Effects of action		X
Action	Goal	X	
	Type	X	
	Normative status	X	
	Intrusiveness		X
	Proportionality		X
	Promptness		X
Responsibility	Obligation or permission	X	
	Distribution	X	X
	Procedure	X	X
	Conflicts	X	X
	Revision	X	X

#### General ethical restrictions on specification

It is beyond the purpose of this paper to propose a particular specification of the precautionary principle as applied to pandemics, for Sweden or more generally. It is also beyond its purpose to take a stand on specific measures such as lockdowns and protective masks. However, I find it necessary to propose certain general ethical restrictions on specification. Specification is not in and by itself a guarantee for ethical soundness. Not all specifications are ethically justified. As Richardson emphasizes in his model of specification, we need to rationally deliberate on the arguments for and against various possible specifications and try reach a well-considered conclusion (Richardson 1990).

A good starting-point is the proposal by Munthe and colleagues in which they point out four basic ethical requirements to be observed in precautionary reasoning regarding pandemics (Munthe et al. 2020 (referring to Hansson 2013)):

- practicability,
- non-arbitrariness,
- proportionality, and
- justification.

Let us take a closer look at these ethical requirements and their implications for specification. They are of vital importance but not without problems.

Practicality is of key ethical significance. This is exactly what specification is all about, namely providing action-guiding content. However, practicality is not merely a matter of presenting a somewhat clearer formulation of the precautionary principle. It is about providing concrete policy options. The specification must be sufficiently precise to present specific policy measures, otherwise it is not ethically satisfying. Moreover, the specification must be feasible. If it is not possible to successfully implement in practice, it does not satisfy the requirement of practicality.

Non-arbitrariness is an essential requirement and specification of the precautionary principle may contribute to satisfying it. Without specification the precautionary principle would remain ambiguous and vague and possible to apply in various arbitrary ways. However, the specification itself must also be non-arbitrary. For example, it should not exclude some options from consideration or focus on uncertainties only for some options. Moreover, the specification must be based on tenable ethical arguments and apply to all situations that are similar, that is, situations that do not exhibit ethically relevant differences. However, non-arbitrariness does not preclude context-sensitivity, that is, consideration of contextual factors. There may be ethically relevant differences among countries, for instance, in terms of different legal and governmental frameworks or different degrees of trust in the Government among the population. Such differences may justify different precautionary measures. Lockdowns, for example, might be justified in some countries but not in others.

As we have seen, proportionality is central to specification of the precautionary principle. Proportionality signifies a balance between magnitude of threat and benefit of action. It is a key goal to strive for, but difficult to attain in practice because the early stages of a pandemic are characterized by uncertainty about the seriousness of threat and uncertainty about the effects of action. If the seriousness of threat is uncertain and the effects of prescautionary measures are uncertain, it is difficult to determine proportionality, especially since the effects of action may include not only public health effects but also broader societal and economic effects. Not only possible good effects of precautionary measures must be taken into consideration but also possible bad effects. Decision-makers may disagree on which degree of intrusiveness of precautionary measures is truly proportional to the degree of seriousness of threat. In a situation of uncertainty some may prefer taking the risk of doing too much, others the risk of doing too little. Moreover, decision-makers may disagree on how to make trade-offs between the benefit of preserving public health and the costs for the intrusiveness of the precautionary measures in terms of, for example, macroeconomic costs, costs for ensuring social order, violation of legal rights such as freedom of movement and children’s right to education. Proportionality requires difficult trade-offs between these various kinds of values and costs.

The final requirement is justification. Justification is necessary for the precautionary principle itself but also for the specification of its various elements and aspects of elements. Ethical justification of proportionality is particularly important, theoretically but also in practical politics. It might be tempting for politicians to introduce more intrusive measures than justified to show that they are truly doing something in a time of severe crisis, as a kind of virtue-signalling political action. But it might also be tempting to introduce less intrusive measures than justified in order to avoid protests. A balance between threat and precautionary measures must be shown by rational argument. There may be disagreement on exactly what the ethical justification of proportionate action should look like. There are different methods of justification: deductive methods (a norm justifies an action), inductive methods (earlier moral experiences of decision-making and action justifies a norm) and coherence methods (reflective equilibrium of a set of norms). In his model of specification Richardson proposes a coherence method. The norm supports and explains the specification and the specification supports and explains the norm (Richardson 1990). All these methods of ethical justification may be possible to apply to precautionary reasoning concerning proportionality. For example, the proportionality may be justified by a utility principle or a rights principle (deduction), by earlier moral experiences of crises and the need for rapid and proportionate action under uncertainty (induction) or by reflective equilibrium between the specification of proportionality, the precautionary principle and other norms within a justice-focused approach (coherence). Although there may be disagreement on the acceptability of these methods, there may still in many cases be a consensus on what to do in practice. If conflict remains on what to do, a majority decision might be an option, although this cannot in itself justify the decision ethically. This can only be done by broader ethical considerations of the value of rapid majority decisions in a situation of serious and urgent crisis.

## Conclusion

In this paper I have taken the Swedish Corona Commission’s critique of the initial Swedish response to COVID-19 as a starting-point for a general analysis of precaution in relation to pandemics. The Corona Commission points out that in contrast to many other countries Sweden did not follow the precautionary principle. Based on this critique, the Corona Commission proposes that the precautionary principle should be included among Sweden’s guiding principles for crisis management.

However, the Corona Commission’s proposal seems theoretically unsatisfying. The debate on the precautionary principle during the last 30 years indicates that the principle comes in many versions and is loaded with problems. In this paper I have focused on one of these problems, namely its lack of clarity. This lack of clarity is illustrated by the difference between the application of the principle by other countries than Sweden during the pandemic and the Corona Commission’s retrospective application of it. While many countries promptly initiated lockdowns based on the principle, the Corona Commission states that Sweden for precautionary reasons should have undertaken various more rapid and intrusive measures but no lockdowns.

I have argued that the problem of lack of clarity is not unsurmountable. The normative status of principles is sometimes misunderstood. A principle is lacking clarity precisely by being a principle and not a rule with a well-defined meaning. A principle suggests a direction but does not prescribe a specific action. To be action-guiding, its content needs to be specified by rational deliberation. In this endeavour I have found Richardson’s ‘model of specification’ useful.

Based on this model, I have presented a framework for specification of the precautionary principle as applied to pandemics. I have focused on the four key elements of the principle (threat, uncertainty, action and responsibility) and presented in detail decisions that need to be made in dealing with its lack of clarity, that is, decisions to eliminate or at least reduce ambiguity and vagueness. I have not proposed any particular specifications. This has been beyond the purpose of the paper. However, I have suggested four general ethical restrictions on specification: practicality, non-arbitrariness, proportionality and justification.
